# Premise typicality as feature inference decision-making in perceptual categories

**DOI:** 10.3758/s13421-021-01240-8

**Published:** 2021-10-08

**Authors:** Emma L. Morgan, Mark K. Johansen

**Affiliations:** School of Psychology, Cardiff University, Tower Building, Park Place, Cardiff, CF10 3AT Wales, UK

**Keywords:** Categorization, Decision-making, Feature inference, Premise typicality, Similarity

## Abstract

Making property inferences for category instances is important and has been studied in two largely separate areas—categorical induction and perceptual categorization. Categorical induction has a corpus of well-established effects using complex, real-world categories; however, the representational basis of these effects is unclear. In contrast, the perceptual categorization paradigm has fostered the assessment of well-specified representation models due to its controlled stimuli and categories. In categorical induction, evaluations of premise typicality effects, stronger attribute generalization from typical category instances than from atypical, have tried to control the similarity between instances to be distinct from premise–conclusion similarity effects, stronger generalization from greater similarity. However, the extent to which similarity has been controlled is unclear for these complex stimuli. Our research embedded analogues of categorical induction effects in perceptual categories, notably premise typicality and premise conclusion similarity, in an attempt to clarify the category representation underlying feature inference. These experiments controlled similarity between instances using overlap of a small number of constrained features. Participants made inferences for test cases using displayed sets of category instances. The results showed typicality effects, premise–conclusion similarity effects, but no evidence of premise typicality effects (i.e., no preference for generalizing features from typical over atypical category instances when similarity was controlled for), with significant Bayesian support for the null. As typicality effects occurred and occur widely in the perceptual categorization paradigm, why was premise typicality absent? We discuss possible reasons. For attribute inference, is premise typicality distinct from instance similarity? These initial results suggest not.

When interacting with complex environments, categories are adaptively important because they enable the classification of novel objects/events and support subsequent attribute inferences for category instances (e.g., that a particular apple is edible). In fact, an important perspective on categories is that their fundamental purpose is to organize information in a way that facilitates attribute inferences. Inference as decision-making in the context of categories has been evaluated in two conceptually related but largely separate research areas—categorical induction and perceptual categorization—each with their own paradigms, effects, and benefits. In overview, the intent here was to establish the existence of effects from the categorical induction paradigm, notably premise typicality, in the more methodologically controlled perceptual categorization paradigm to be better able to test hypotheses about the, as yet unclear, mental representations underlying these effects.

Categorical induction involves making judgements about unknown features of category instances based on features of known instances, usually for real-world categories (e.g., inferring an instance is edible because other apples have been; Feeney et al., 2007; Gelman & Markman, 1986; Heit, 1998, 2000; López et al., 1992; McDonald et al., 1996; Medin et al., 2003; Medin et al., 1997; Osherson et al., 1990; Proffitt et al., 2000; Rips, 1975, 2001; Sloman, 1993; Smith et al., 1993; Tenenbaum et al., 2006). Research in this paradigm has assessed what properties affect these inferences, ordinarily by using judgements about arguments. A formal example of a categorical induction argument is, “Sparrows have property X Therefore Geese have property X” (Hayes et al., 2010). This argument starts with a known instance of the Category Birds, sparrows, but attaches an unknown (commonly blank) attribution to it, property X. The argument structure implies a generalization of an unknown attribute, property X, from one known category member to the other, sparrows to geese. The common response measurement in this paradigm is a rating of the likelihood of the conclusion being true (geese have property X) given that the premise is true (sparrows have property X). So, these likelihood ratings measure the argument strengths for attribute inferences.

The categorical induction paradigm has a corpus of well-established empirical effects in terms of influences on judged argument strength (summarized in Hayes et al., 2010; Heit, 2000; Osherson et al., 1990). Of these effects, arguably the most important is premise typicality, described below. However, premise conclusion similarity will also be important here. Other effects include premise diversity, in which having more diverse category members make stronger arguments, and premise numerosity, in which having more premises makes for stronger arguments.

The premise typicality effect is the finding that arguments about generalizing a feature based on a typical premise (i.e., using a typical category member) are judged to be stronger than arguments based on an atypical premise, using an atypical category member (Carey, 1985; Hayes et al., 2010; Osherson et al., 1990; Rhodes et al., 2008; Rips, 1975). For example (Hayes et al., 2010), “Sparrows have property X Therefore Geese have property X” is judged to be a stronger argument than “Penguins have property X Therefore Geese have property X.” The first argument is judged as stronger because a sparrow is a more typical instance of the bird category than a penguin and shares more features with other category members.

The premise conclusion similarity effect is that the more similar the premise instance is to the conclusion instance, the stronger the argument (Gelman, 1988; Hayes et al., 2010; Osherson et al., 1990; Rips, 1975). For example (Hayes et al., 2010), ‘Leopards have property X Therefore Lions have property X,’ is judged stronger than, ‘Leopards have property X Therefore Koalas have property X,’ because leopards are more similar to lions than to koalas.

In this paradigm, the effects of premise typicality and premise conclusion similarity are treated as distinct because tests of premise typicality usually attempt to control for the similarity between the premise and conclusion instances. Similarity has been commonly assessed using judgments for pairs of instances to create a (low dimensional) similarity space using multidimensional scaling in which specific category instances are imbedded. Similarities between instances in the space then correspond (inversely) to their distances, the smaller the distance the more similar (Rips et al., 1973). However, the underlying bases for these similarities between instances is not particularly clear, in part because the instances of real-world categories have many complex attributes and relationships (e.g., the similarities between geese and sparrows versus geese and penguins in the example above). Because the instances of real-world categories are not manipulated in terms of the complex attributes they share, it is difficult to know how they contribute to similarity or how well similarities between them have been controlled or interact with inferences. At minimum, directly manipulating the shared attributes of category instances seems likely to facilitate controlling their similarities more strongly.

Even more fundamentally, assessing the mental representations underlying categorical induction effects at the level of instance attributes is not straightforward (e.g., how is a lion represented in terms of its attributes?). This is in part because of the complexity of the attributes and the elaborate web of prior knowledge that concepts are embedded in (e.g., the reasons lions and leopards are similar). Establishing categorical induction effects in the perceptual categorization paradigm would allow a more direct assessment of the category representations underlying these effects using the well-specified representation models largely developed using the perceptual paradigm. These include prototype models (Homa et al., 1981; J. D. Smith, 2002; J. D. Smith & Minda, 2001), based on an abstracted typical instance (the prototype) composed of typical features, and exemplar models (Kruschke, 1992; Medin & Schaffer, 1978; Medin & Schwanenflugel, 1981; Nosofsky, 1986; Nosofsky & Johansen, 2000; Nosofsky & Zaki, 1998), based on many stored instances and their configurations of features. Although the representations are different, both kinds of models categorize new instances based on their similarities to the representations of known categories.

Many of the categories people learn and use are based, at least in part, on perceptual properties of instances with attached conceptual labels (e.g., cat, tree, cloud). And, as discussed above, learning such sophisticated categories involves a complex interplay with prior knowledge making it difficult to assess the representational basis of these categories, how people learn and use them, because so much is unknown or hard to characterize. To control for these complexities, the perceptual categorization paradigm uses novel, carefully constrained stimuli and newly constructed categories as a way to assess the basic mechanisms of category learning and decision-making(e.g., Griffiths et al., 2012; Honke et al., 2016; Johansen & Kruschke, 2005; Love, 2002; Medin & Schaffer, 1978; Medin & Schwanenflugel, 1981; Nosofsky & Zaki, 2002; Shepard et al., 1961; Yamauchi & Markman, 1998; Zeigler & Vigo, 2018). So, the paradigm facilitates evaluating how people represent new concepts and make inference decisions using those concepts by simplifying and controlling the categories and feature instances. In particular, these constructed perceptual categories (e.g., Fig. 1) have the important property of allowing similarities between category instances to be directly manipulated in terms of sharing a small number of simple features/attributes. And this strongly specified similarity information in terms of shared features can be given to representational models and used to draw conclusions about what category representation a person was using by fitting the models to their categorization data.

**Fig. 1:**
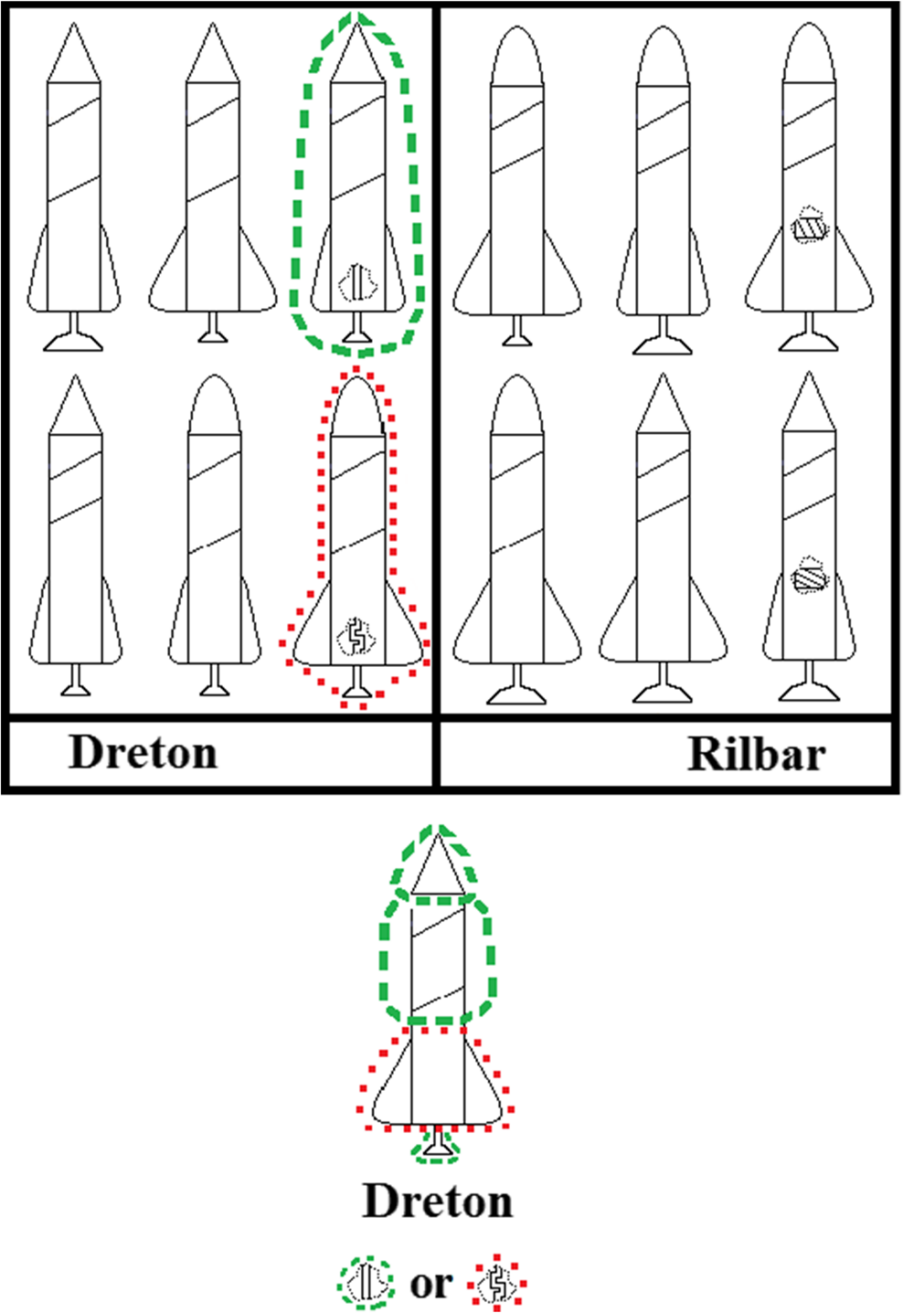
An example of an “ordinary” premise typicality trial used in Experiments 1 and 2, with the category summaries above a test instance. The individual rocket ship at the bottom of the figure is a premise typicality testing case: a rocket without a hidden feature, presented with its category label and two hidden feature response options. Typical features/instances are indicated by green dashed outlines and atypical features/instances by red dotted outlines, added for explanatory purposes only. Participants did not see these outlines. (Color figure online)

Critically, given the present focus on premise typicality effects in categorical induction, constructed perceptual categories have been widely shown to induce typicality effects like those observed in real world categories where some instances are better instances of categories than others, are categorized more accurately and so forth (Ashcraft, 1978; Holmes & Ellis, 2006; McCloskey & Glucksberg, 1978; Rosch et al., 1976; Rosch & Mervis, 1975). In particular, Rosch and Mervis (1975) explained typicality in terms of features shared across category instances: a category instance is most typical when it has many features in common with other members of the same category and few features in common with members of other categories. For example, a robin is a more typical member of the category “bird” than a penguin is, in part because a robin shares the very common attribute that it flies with many other birds while a penguin does not. The perceptual categorization paradigm is particularly suited to setting up this kind of feature sharing in very controlled ways and has widely demonstrated typicality effects (Bourne, 1982; Johansen, Fouquet, Savage, & Shanks, 2013; Medin & Schaffer, 1978; Posner & Keele, 1968; Rosch et al., 1976; Rothbart & Lewis, 1988), perhaps most notably in support for prototype models of category representation. While exemplar representation—categorization based on similarity to known category instances—has been successful in accounting for typicality effects (Kruschke, 1992; Medin & Schaffer, 1978; Nosofsky, 1988, 1991), prototype representation is a more direct embodiment of typicality in terms of classification based on similarity to an abstracted best, average instance of the category, the prototype (Bourne, 1982; Homa et al., 1981; Richards & Chiarello, 1990; Rosch & Mervis, 1975; J. D. Smith, 2002). Given the prevalence and importance of typicality effects, prototypes seem to provide a compelling basis not only for category representation but particularly for attribute decision-making based on categories, the focus of the categorical induction paradigm. As summarized by Murphy (2002), “If read literally, almost all the work on category-based induction takes a prototype view of concepts” (p. 265). Attribute feature inference in relation to a category prototype is intuitively compelling (e.g., inferring a new instance of the bird category will fly is sensible because the typical bird, say a robin, flies). Ultimately, establishing premise typicality in the perceptual paradigm would facilitate a direct comparison of these possible representations underlying feature inference.

The strength of attribute judgments in categorical induction and feature inference in perceptual categorization are similar as both use category membership for making decisions about instance properties. Both are based on using category knowledge to make inferences about what feature a category instance might have, but the origin of the knowledge is usually different. Categorical induction normally uses known categories such as birds or mammals which are complex real-world categories acquired over a lifetime (Heit, 1998; McDonald et al., 1996; Medin et al., 2003; Osherson et al., 1990; Rips, 1975; Sloman, 1993; E. E. Smith et al., 1993; Tenenbaum et al., 2006). Feature inference commonly uses newly learned, constructed categories as the basis for making attribute choices (e.g., Griffiths et al., 2012; Johansen & Kruschke, 2005; Murphy & Ross, 1994; Yamauchi et al., 2002; Yamauchi & Markman, 1998). But summarized, rather than learned, presentations of constructed category instances have also been used to evaluate category-based feature inference (e.g., Griffiths et al., 2012; Johansen et al., 2015; Murphy & Ross, 1994, 2010; Yamauchi & Markman, 2000; Yamauchi & Yu, 2008). This category summary approach using visually presented sets of category instances is much like the presentation of summarized verbal information in the categorical induction paradigm (e.g., Robins have property X) and is the approach used in the present experiments.

Categorical induction judgements and feature inference in perceptual categorization are similar as both use category membership for making decisions about instance properties; both ask participants to decide about an instances’ attribute/feature that is not visible. However, the nature of these responses is usually different, a rating of argument strength in categorical induction versus a chosen feature in feature inference. Nevertheless, these should be related: If a participant believes that one argument is stronger than the other as manifested through a difference in ratings on the likelihood scales, the participant should plausibly choose the response/feature associated with the stronger argument when given a forced choice between possible features. Overall, the commonalities between these two paradigms suggest that effects found in the categorical induction paradigm should also occur in the more methodologically controlled perceptual categorization paradigm—notably, premise typicality effects, allowing a more direct assessment of the category representations underlying these effects.

To investigate the premise typicality effect via feature inference in the perceptual categorization paradigm, the following experiments used visual summaries of instances from constructed categories (Fig. 1) that had two crucial properties necessary to be able to test the effect—a typicality structure, based on family resemblance, and attached hidden features. First, the categories needed to contain instances with different levels of typicality. At least one instance needed to have a higher level of typicality than others (the instance outlined by a green dashed line in Fig. 1) and another instance needed a lower level of typicality (the instance outlined by a red dotted line) so as to correspond to the typical and atypical premises in the basic test of premise typicality. Second, these instances needed “hidden”/not always visible features indicated by x-ray outlines (Fig. 1) attached to serve as response options that test for a preference to generalize the typical feature more than the atypical as in categorical induction, e.g., “Robins have property X.”

The present experiments used family resemblance structures which have been regularly used in perceptual category learning because real-world categories commonly have family resemblance structures with common features shared by many but usually not all instances (Love, 2002; Markman & Maddox, 2003; Minda et al., 2008; Rosch & Mervis, 1975; Ward et al., 1990). The family resemblance structure in the following experiments had a reasonably strong typicality gradient which included a prototype, consisting of all (four) typical category features, a set of instances that differed from the prototype by one atypical feature and a very atypical instance that differed from the prototype by having two atypical features (Table 1). In the table, each row specifies a particular category instance with six instances in each category, Category A and Category B. The 1 and 3 values on each dimension represent the two possible values each feature dimension could have: wide/narrow wings, long/short body band, large/small booster, and pointed/rounded cone shape. For Category A, the most common feature on every dimension is feature 1 and for Category B the most common value is 3, so these are the typical features, whereas 3 for Category A and 1 for Category B are the atypical features. So, the most typical category instance, the prototype, had all typical features (e.g., A1111 in Table 1). Four “ordinary” typicality instances differed from the prototype by one feature (e.g., A3111) and the atypical instances differed from the prototype by two features (e.g., A3113). So, for example, the Dreton category prototype (A1111, the rocket outlined in green dashes in Fig. 1) had features typical of a Dreton—in this case, a long body band, small booster, pointed cone, and narrow wings. The atypical instance (A3113, the rocket outlined in red dots in Fig. 1) had two features typical of the Dreton category—a long body band and small booster, and two atypical features, a rounded cone, and wide wings. So, this category structure has the typicality gradient necessary for testing premise typicality.

**Table 1:**
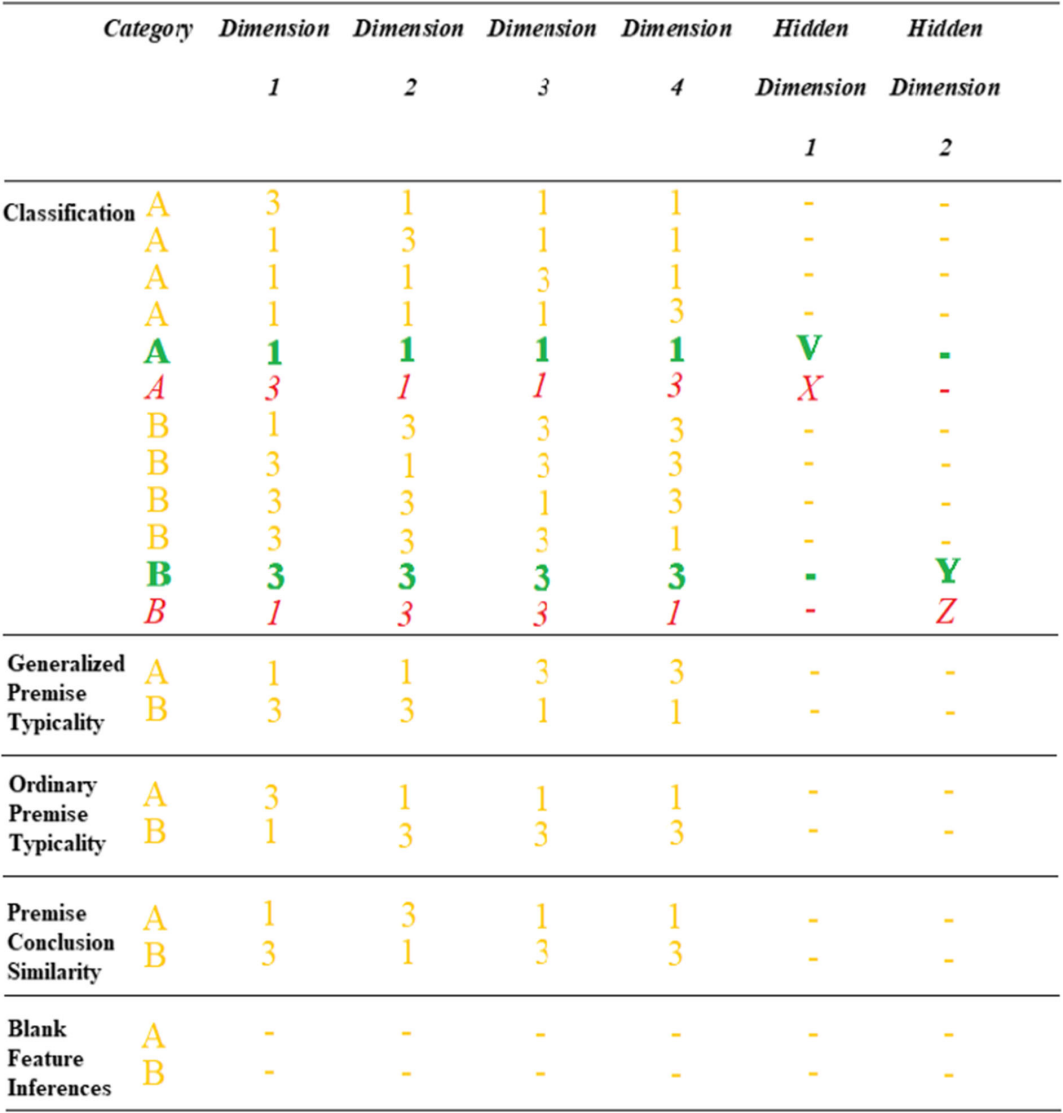
The abstract category structure of the category summary (classification) and key test cases for Experiments 1 and 2

*Note.* The full abstract specification of all testing trials is in Appendix A. Text attributes indicate the typicality structure of the classification category instances: bold green = typical, italic red = atypical, regular font yellow = ordinary category instances). Dashes indicate the absence of a feature on a given dimension, see main text for an explanation of the testing trials

In addition, the typical and atypical category instances had hidden features attached to each (V, X, Y, and Z in Table 1) as indicated by dotted cut-outs revealing the interior hidden features which were the straight/curved pipes and vertical/horizontally lined boxes in Fig. 1. These cut-outs were intended to convey the hidden nature of these features by allowing participants to “see into” the typical and atypical rocket ships while also suggesting that the other rocket ships might have these features but that they were currently hidden due to the lack of cut-outs. So, a feature inference task tested premise typicality with the structure in Table 1 by attaching a hidden feature to the prototype (typical) instance (e.g., A1111V) and to the atypical instance (e.g., A3113X) for each category. Participants were asked which hidden feature should be attached to a test instance (e.g., A3111?) that did not (yet) show an attached hidden feature. Critically, the test instance shared an equal number of features, three, with the typical and atypical instances (e.g., Fig. 1). So, a premise typicality effect in this paradigm corresponds to a preference for the feature associated with the typical instance (e.g., the straight pipe in Fig. 1) over the atypical instance (the curved piped). One assessment of premise typicality involved test cases that were “ordinary” instances of the category as they were included in the category summary (e.g., A3111 in Table 1). The other main assessment was a “generalized” premise typicality test using new category instances that were not in the summary (e.g., A1133 in Table 1). Finally, a conceptually weaker assessment of the effect presented a test instance with no perceptual features at all and only a category label, the “blank” feature inferences in Table 1. Finally, Fig. 2 summarizes the mapping between premise typicality effects in the two paradigms.

**Fig. 2:**
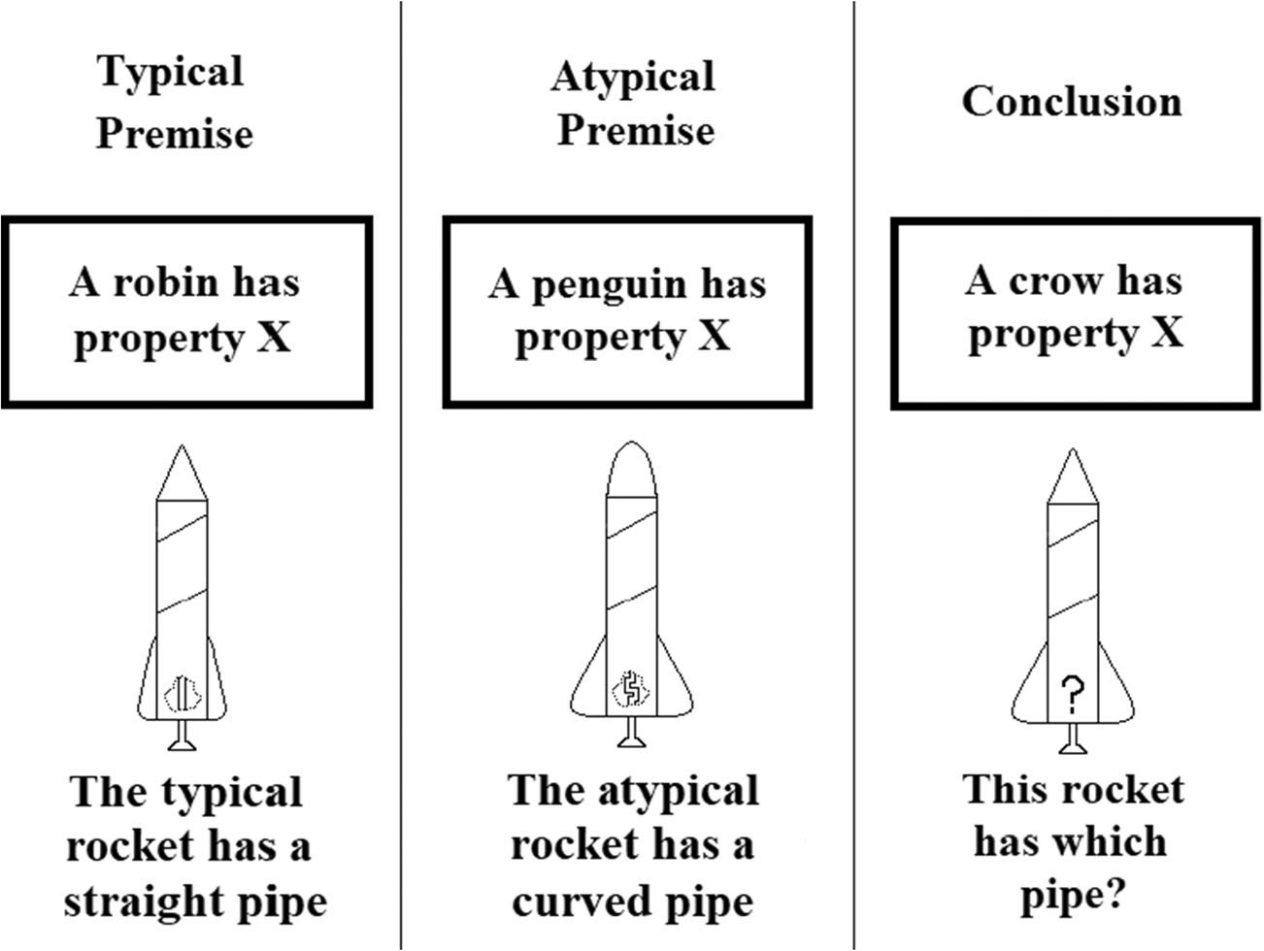
A summary of the mapping between premise typicality in the classic categorical induction paradigm as linguistic descriptions and in the perceptual categorization paradigm as perceptual rocket ships. Note that the participants did not see the phrases “The typical rocket has a straight pipe”; “The atypical rocket has a curved pipe”; these were added to the figure for explanatory purposes only

The present experiments also tested premise conclusion similarity using trials where the test instance was more similar to the typical category instance than to the atypical instance, and participants chose between the hidden feature attached to the typical versus atypical instance. For example, the testing trial A1311 in Table 1, has three features in common with the typical instance for Category A, A1111, and only one feature in common with the atypical instance, A3113. A preference for the typical hidden feature on this test would correspond to a premise typicality *like* effect that is confounded with similarity as it can be based on similarity rather than typicality.

In overview, both experiments tested for premise typicality effects using visual category summaries of category instances (as shown in Fig. 1) that were present during all of the key testing trials. Experiment 1 was a pure decision-making task in the sense that participants received no feedback about the correctness of their responses on any trials. In contrast, Experiment 2 had an initial training phase where participants were told whether or not their answers were correct on some trials, but again, the category summary was available throughout. The intent of both experiments was to replicate the premise typicality effect in the perceptual paradigm so as to be able to clarify the representational basis of the effect and of feature inference more generally.

## Experiment 1

To test premise typicality using perceptual categories, the key attributes of the categories include a typicality structure and attached hidden features. So, key prerequisites for premise typicality include participants showing sensitivity to both the typicality structure and to the attachment of the hidden features to that structure. Before and after the key tests of premise typicality, classification testing trials for all category instances assessed participants’ sensitivity to the typicality structure (Table 2) both with and without the hidden features present. In addition, testing trials queried which hidden features were attached to the typical and atypical category instances in the same blocks of trials that tested premise typicality (Table 2) and also in separate blocks. After the key tests of premise typicality, participants then inferred regular (nonhidden) features for category instances. Some of these inferences were for “exception” features, that is atypical features, for example, participants were asked to infer the missing feature for A?113X which only perfectly matched one instance in the summary, the atypical instance A3113X. Other feature inferences were ambiguous in that the test case (e.g., A?111) matched two different category instances in the summary, A3111 and A1111. Finally, the very end of this experiment included tests of some common categorical induction effects from the standard categorical induction paradigm including premise typicality based on verbal statements (e.g., “Sparrows have property X Therefore Geese have property X”) with argument strength judgments.

**Table 2:**
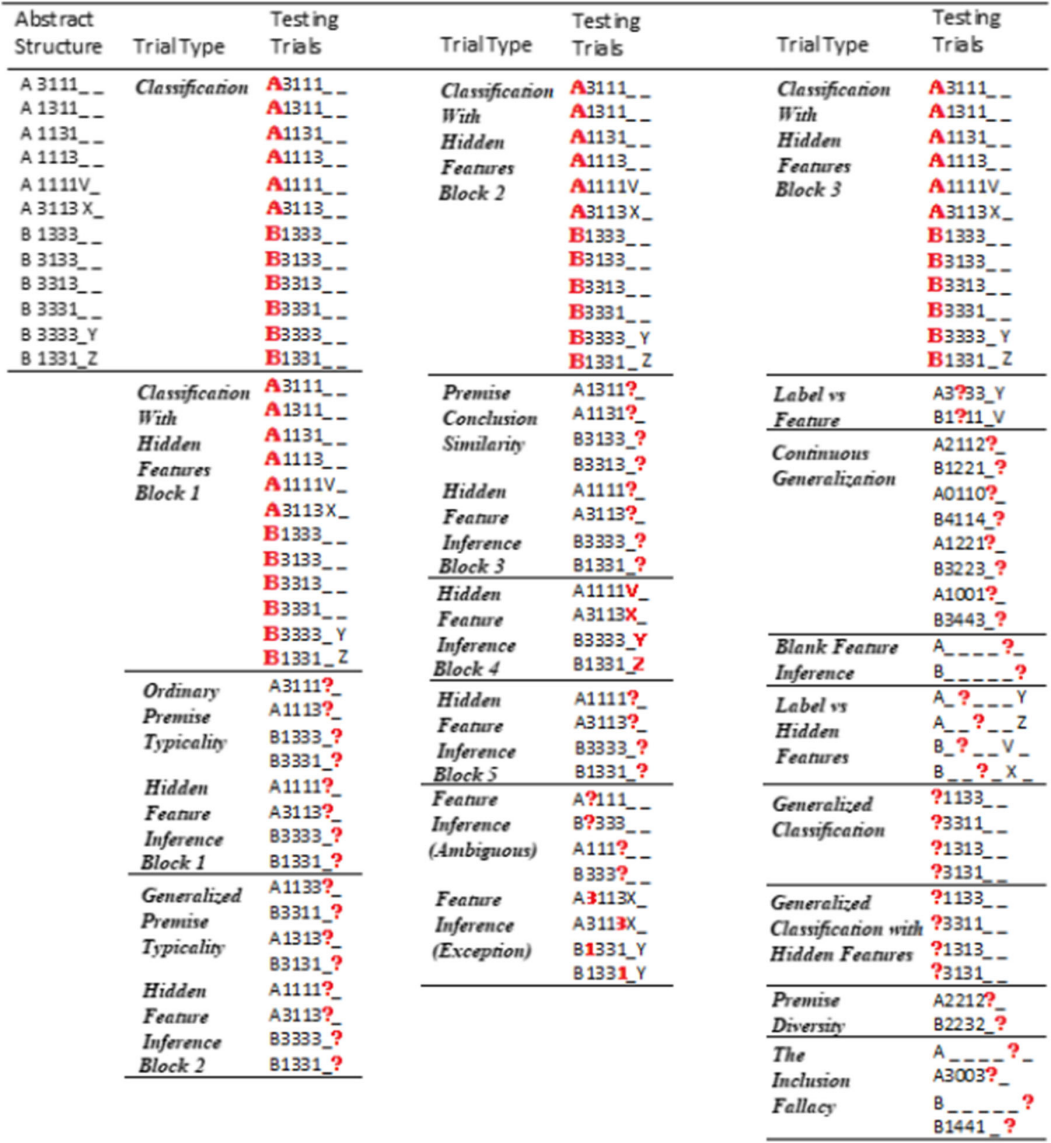
The abstract structure of the categories and all testing trials in Experiment 1

*Note.* Category labels/features in bold red for a given test case were queried and had an unambiguous correct answer in the category summary. Question marks in test cases indicate a queried feature that did not have a clear correct answer (i.e., with no single best match in the category structure). See main text and Appendix B for all testing block descriptions

### Materials and methods

#### Participants

Forty-eight Cardiff University students participated for course credit or payment.

#### Materials and procedure

The rocket ship stimuli in this experiment (Fig. 1) varied on four binary valued dimensions; wing width (wide/narrow), body band height (long/short), booster size (large/small), and nose cone shape (pointed/rounded). In addition, there were two hidden feature dimensions indicated by dotted cut-outs, pipes (straight/curved), and boxes (with horizontal/vertical lines; see Fig. 1).

The assignment of the four physical stimulus dimensions in Fig. 1 to the four abstract dimensions composing the category instances (Table 2) was chosen randomly for each participant, as was the assignment of the two hidden features dimensions and their abstract values. Similarly, the category labels Dreton and Rilbar were assigned randomly to the two abstract categories (A and B in Table 1) for each participant.

Testing trials included a category summary on the computer screen above the testing item consisting of twelve rocket ships with their category labels underneath (e.g., Fig. 1). On all trials participants chose between two on-screen response options, either the category labels or two different features, below the summary by mouse clicking the chosen option. After each trial, participants rated their confidence from 1 (*very unconfident*) to 9 (*very confident*).

Within each testing block (Tables 1 and 2), the order of trials was determined randomly for each participant. Testing blocks included tests of instance classification, hidden feature attachment, premise typicality, premise conclusion similarity and various features inferences of nonhidden features. See the Introduction for a detailed description of the key tests. As well as the classification testing trials, there were 62 feature inference testing trials (see Table 2). The experiment also included additional tests toward the end (Table 2) that are not central to the key arguments presented here, including tests contrasting labels versus nonhidden features and labels versus hidden features, and continuous feature dimension tests, as described in Appendix B.

The category summary, composed of the 12 category instances at the top of Table 2, was present on every testing trial (e.g., Fig. 1). The summary included attached hidden features for the typical and atypical instances except in the first block (Classification, Table 2), the general classification block and the last two blocks (Table 2). At the end of the experiment, 10 classic paradigm categorical induction effect questions using real-world categories tested the standard versions of premise typicality and other common effects (listed in Appendix C and adapted from Hayes et al., 2010). Participants made argument strength ratings on a scale from 1 (*very unconfident*) to 9 (*very confident*).

Participants first read through the on-screen instructions then proceeded through the 118 test trials (Table 2) and 10 classic paradigm categorical induction effect questions, Appendix C. The experiment took about 30 minutes.

#### Analysis

Our analytic approach is to report Bayesian statistics in parallel with the more familiar null-hypothesis significance tests. The primary motivation for reporting Bayesian statistics is that, unlike null hypothesis significance testing, Bayesian statistics provide a straightforward way of demonstrating significant support for the null hypothesis of no difference. (In contrast, null hypothesis significance testing is framed in terms of rejecting the null hypothesis rather than in terms of a straightforward potential to support it.) Bayesian statistics are commonly reported as a “Bayes Factor” in terms of a ratio of support for the alternative hypothesis to the support for the null hypothesis, usually symbolized as BF_10_. A common interpretation (Jeffreys, 1961) is that a Bayes Factor larger than 3 indicates substantial support for the alternative hypothesis over the null, and a Bayes factor less than 1/3 = 0.333 indicates substantial support for the null hypothesis. The units of analysis for most of these results was a proportion across test trials of a given type (see Table 2) for a given participant (e.g., classification accuracy was evaluated as proportion correct across classification trials).

In overview, the results sections are organized in terms of first presenting assessments of necessary preconditions for a valid assessment of premise typicality: classification accuracy, to demonstrate awareness of the category typicality structures, and hidden feature inference, to demonstrate attachment of hidden features to instances in that structure. These are followed by the three different tests of premise typicality which are then contrasted with the results for premise conclusion similarity.

### Results and discussion

**Fig. 3:**
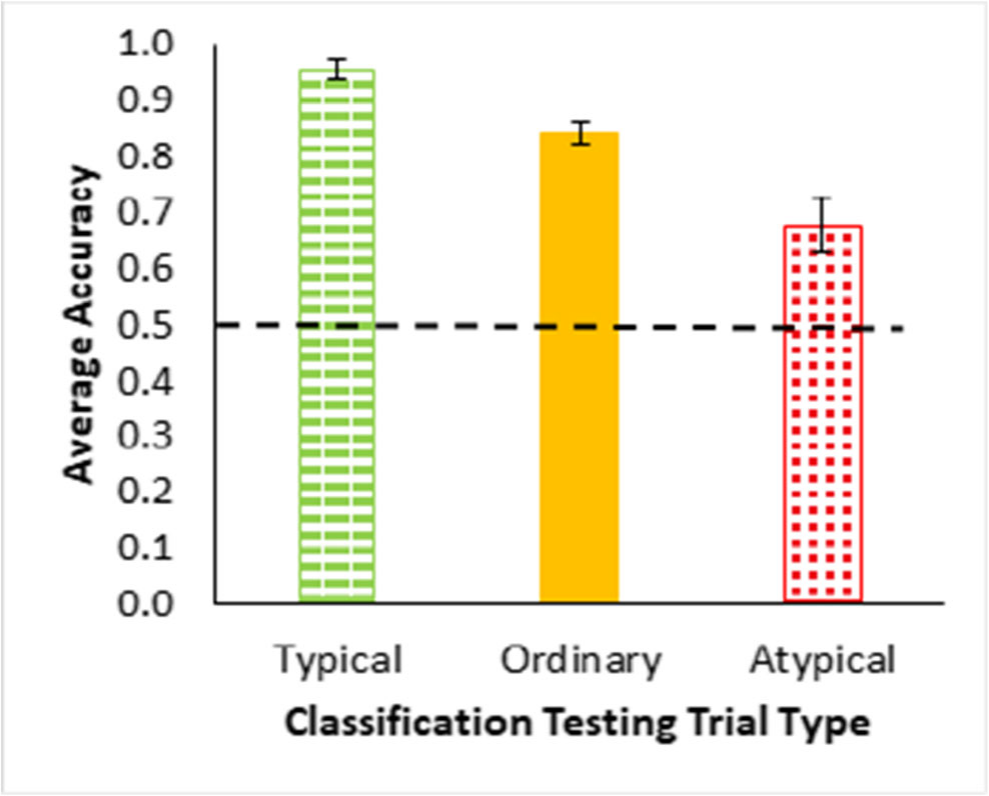
Averaged accuracy as proportion correct for all classification testing trials in Experiment 1, grouped by trial type—typical = green dashes, ordinary = yellow, atypical = red dots (Table 1). The large dashed line is a reference for two-option chance responding. Error bars show ±1 standard error

The classification test results (Fig. 3) show a typicality effect in terms of higher accuracy for more typical than less typical instances, a necessary precondition for assessing premise typicality, *F*(1.17, 54.951) = 28.44, *p* < .001, ${\eta}_{partial}^2$ = 0.377, based on proportion correct across all classification trials for each participant by type. Note for this single factor within-participants analysis of variance (ANOVA) that the assumption for sphericity was likely violated and the Greenhouse–Geisser correction applied to the degrees of freedom. The Bayes factor for these results was BF_10_ = 7.778e+7 indicating that the ratio of support for the alternative hypothesis of differences in typicality over the null hypothesis (no differences) substantially favored the alternative hypothesis in terms of being substantially greater than 1. Bayesian statistics are from JASP using the default Cauchy prior (JASP Team, 2019). All individual testing trial averages for the cases in Table 2 are reported in Appendix A.

**Fig. 4:**
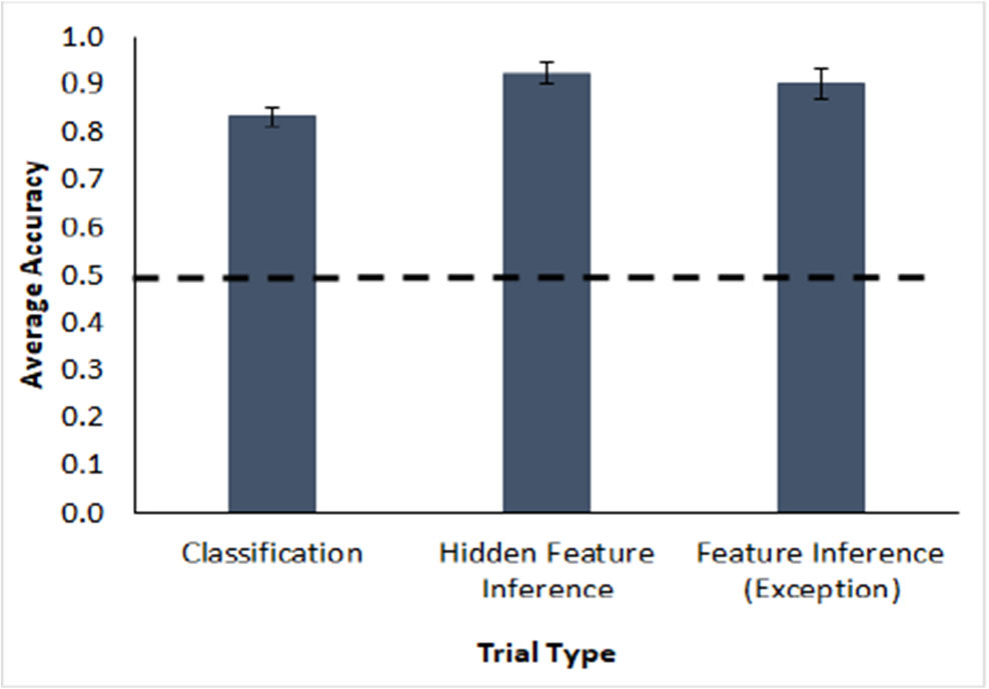
Average accuracy as proportion correct for classification, hidden feature inference and feature inference (exception) testing trials in Experiment 1(Table 2). The dashed line is a reference for two-option chance responding. Error bars show ±1 standard error

The hidden feature inference trials (Figure 4 middle bar) showed good, significant attachment of the hidden features to the typical and atypical instances, also a necessary precondition for assessing premise typicality, *t*(47) = 20.2, *p* < .001, a single-sample*t* test against a mean of 0.5, *d* = 2.9, BF_10_ = 4.163e+21. Additionally, overall classification performance was good (Fig. 4, left bar), *t*(47) = 16.2, *p* < .001, single-sample*t* test against 0.5, *d* = 2.3, BF_10_ = 6.005e+17, as was feature inference (exception) performance, *t*(47) = 12.8, *p* < .001, single sample against 0.5, *d* = 1.8, BF_10_ = 9.221e+13. Taken together, these results indicate that participants engaged with and understood the instances in the category summary.

So, in summary, participants showed key attributes conceptually necessary as prerequisites for a premise typicality effect: good engagement in terms of accurate classification of category instances, apparent sensitivity to the typicality structure of the categories with some instances more typical than others and good attachment of hidden features to the typical and atypical instances.

**Fig. 5:**
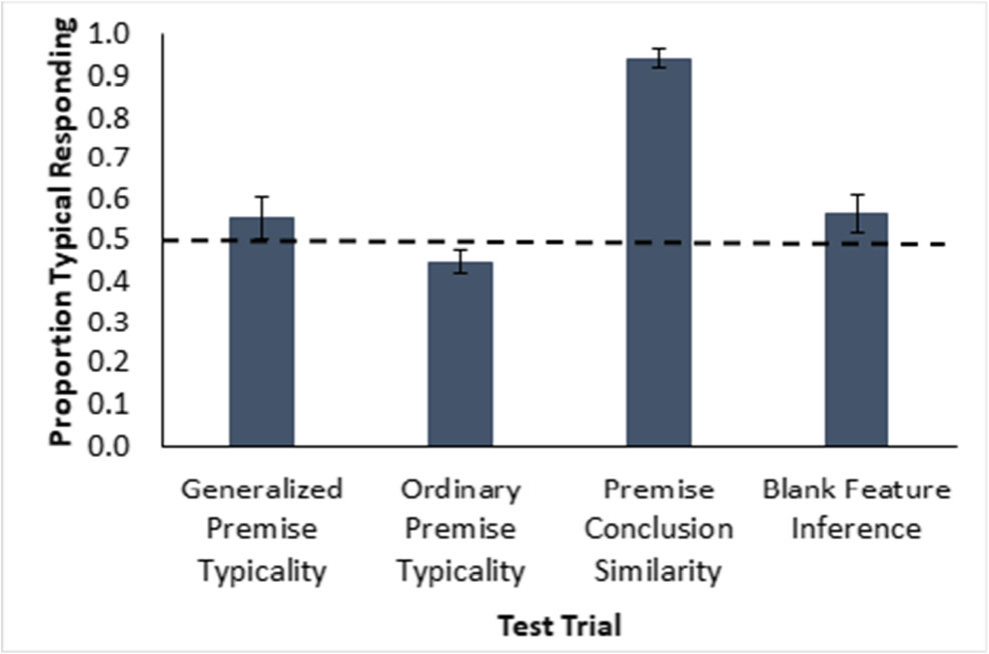
Average proportion of typical hidden feature responding for generalized and ordinary premise typicality, premise conclusion similarity and blank feature inference in Experiment 1(Table 2). The dashed line is a reference for two-option chance responding. Error bars show ±1 standard error

Despite a typicality effect and hidden feature attachment, no premise typicality occurred (Fig. 5) on any of the three distinct tests of plausible ways it might have occurred (based on the proportion of typical hidden feature responses for trials of a given type): not on the generalized premise typicality trials with generalization tests different from the category instances, *t*(47) = 1.0, *p* = .312, single-sample*t* test evaluated against 0.5, *d* = 0.15, BF_10_ = 0.256, note substantial Bayesian support for the null hypothesis, or on the ordinary premise typicality trials based on known category instances; *t*(47) = −1.9, *p* = .067, *d* = −0.3, BF_10_ = 0.783, note, this Bayes factor is in the direction of atypicality. And finally, blank trials with only the category label present (and so with no influence of feature similarity) also did not show a significant preference for the typical hidden feature, *t*(47) = 1.4, *p* = .182, *d* = 0.2, BF_10_ = 0.368. Overall participants showed no preference for generalizing the hidden feature attached to the typical instance compared with the atypical instance when similarity was controlled (i.e., no premise typicality effects).

Despite the absence of premise typicality, participants were not simply without preference between hidden features as a significant premise conclusion similarity effect did occur in terms of a preference for the typical hidden feature over the atypical hidden feature, *t*(47) = 20.7, *p* < .001, single-sample*t* test against 0.5, *d* = 3.0, BF_10_ = 1.216e+22, based on the proportion of typical feature responses across testing trials. And this proportion of typical responding was significantly higher than for generalized premise typicality, *t*(47) = 6.7, *p* < .001, a within-participant*t* test, *d* = 1.0, BF_10_ = 512857. So, a premise typicality *like* effect occurred here but only when typicality was confounded with similarity (i.e., the test item was more similar to the typical instance than the atypical one; see Table 1).

**Fig. 6:**
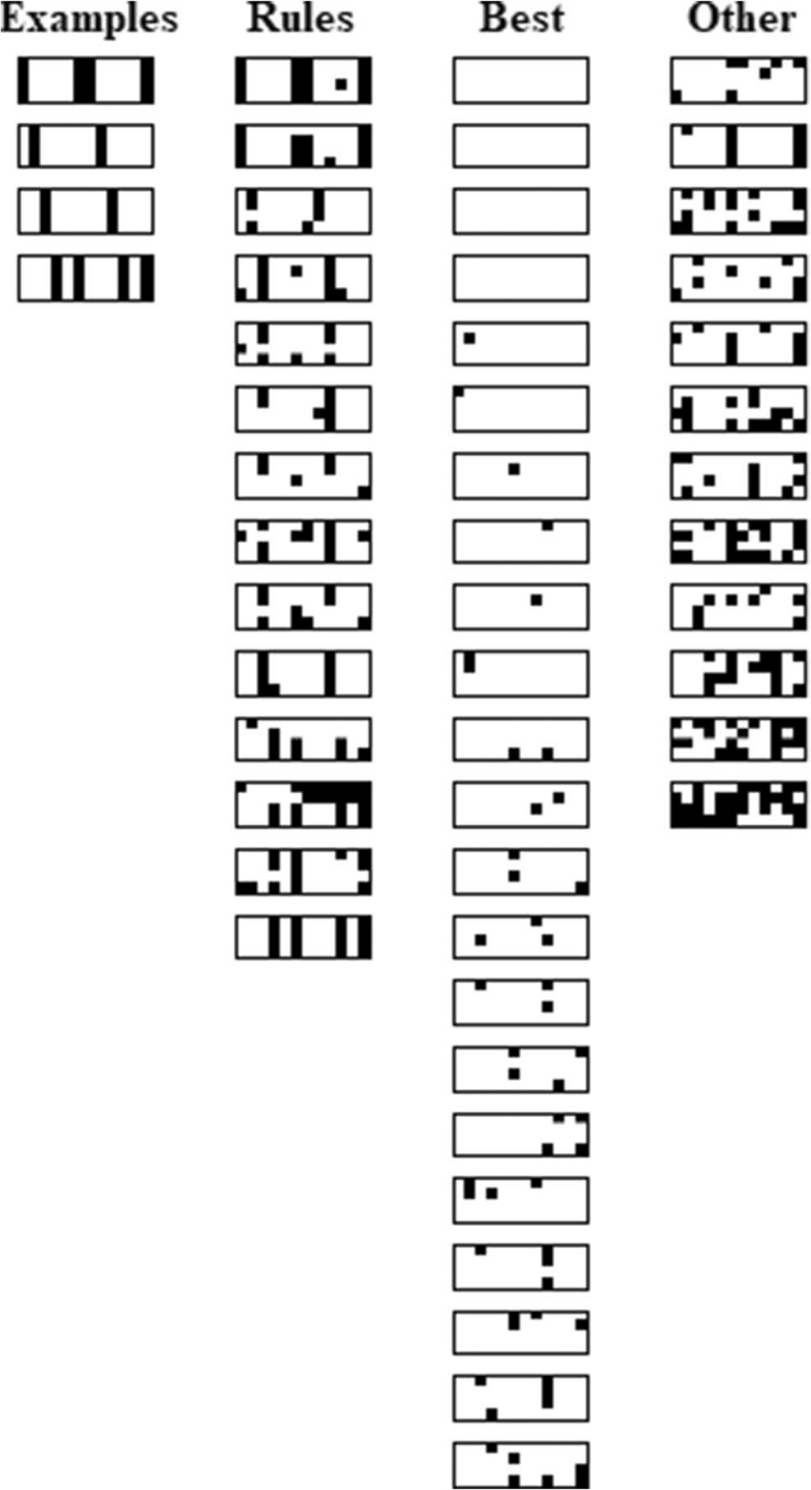
Error diagrams showing performance of each individual participant over classification testing trials for the category summary instances in Experiment 1. Instances are arranged in columns (ordered as in Table 1) and testing blocks are arranged in rows. See main text for details. Black dots = incorrect answers, white “dots” = correct answers. The “examples” grouping shows error patterns corresponding to unidimensional rules in order, with a dimension one rule at the top and a dimension four rule at the bottom. The “rules” grouping has apparent suboptimal dimensional rule users, the “best” group includes high accuracy performers, and the “other” group has the remaining participants that used various other strategies

To clarify the strategy used by each participant, error diagrams show all individual participant responses to summary instance classification tests (Fig. 6). Each rectangle shows a given participant’s responses where black dots represent incorrect answers on individual trials and the remaining white space (i.e., white “dots”) represent correct answers on individual trials. Each rectangle is made up of 12 columns which specify the classification trials for all 12 summary category instances (ordered as in Table 1) and four rows which indicate performance on each instance over the four classification testing blocks. The first column of four rectangles labelled, “Examples,” indicates the pattern of responding consistent with a unidimensional rule, respectively, on each of the four feature dimensions (e.g., a rule on dimension one would be “a [1 feature] indicates [Category A], a [3 feature] indicates [Category B]”). Using this rule corresponds to errors on instances A3111, A3113, B1331 and B1333 (Table 1) and these exceptions to the rule can be seen as vertical black lines of errors in the diagrams. Subsequent columns of rectangles represent participants grouped by performance. The first grouping has participants who responded consistent with one of the four dimensional rules (29% of participants), the second grouping has participants with the best overall accuracy (46% of participants; i.e., participants who were not apparently using a rule and made six or fewer errors), and the third grouping has participants whose responding did not correspond to either of the other groups (25% of participants). So, a substantial number of participants who engaged with the task seemed to use dimensional rules. The potential relationship of this to the apparent typicality effects is discussed below.

The classic categorical induction tests showed a significant effect of premise typicality based on a difference in rated argument strength for the typical premise (mean = 4.63) greater than the atypical premise (mean = 3.46), *t*(47) = 4.7, *p* < .001, a within-participant*t* test, *d* = 0.7, BF_10_ = 1011.376. This replication of premise typicality in the classic paradigm suggests that the observed absence of premise typicality in the perceptual paradigm was not due to a defect in the participant population.

The proportion of participants who were apparently using unidimensional rules (Fig. 6) provides a possible explanation for the observed absence of a premise typicality effect here. Rule-based performance could give rise to a pseudo-typicality effect as a result of averaging across participants without individual participants having any appreciation of the typicality structure. Stated abstractly, a rule chosen on the basis that a 1 on a dimension belongs to Category A and a 3 belongs to Category B will correspond to accurate classification of the typical instances (Table 1). However, each unidimensional rule would cause errors in classifying two ordinary instances, somewhat reducing accuracy for these compared with the typical instances. And two out of the four unidimensional rules would cause additional errors on the atypical instances, reducing accuracy even further compared with the typical instances. Therefore, an apparent typicality effect could occur even if participants were classifying instances using unidimensional rules. So, a subset of participants was apparently using dimensional rules providing at least a partial explanation for the observed lack of premise typicality and suggests modifying the task to encourage a wider distribution of attention across dimensions.

## Experiment 2

The purpose of this experiment was to more strongly motivate participants to attend to all stimulus dimensions to produce a strong and well-founded appreciation of the category typicality structure. Regehr and Brooks (1995) found that the use of a category summary produced single dimensional sorting of instances into categories which is analogous to unidimensional rule use in Experiment 1. So, a widely found preference for unidimensional rule sorting (Medin et al., 1987) is likely related to a tendency to use unidimensional rules in other tasks. Lassaline and Murphy (1996) found that a way to encourage family resemblance sorting (and therefore encourage an appreciation of typicality) was to have participants undergo a task before sorting that facilitated noticing the relationships between instances and features. They found that initially making feature inferences subsequently encouraged more family resemblance sorting compared with control conditions. At minimum, this suggests that feature inferences are a good way to get participants to attend to all the features in the category instances. An initial task that encourages attention to multiple dimensions should reduce the number of participants who show a tendency to use unidimensional rules while increasing the appreciation of the category typicality structure across multiple dimensions. To encourage participants to use all of the feature dimensions in premise typicality decision-making, the current experiment first presented a feedback learning task based on the category summary that included feature inference trials on each feature dimension composing the category instances in Table 3.

**Table 3:**
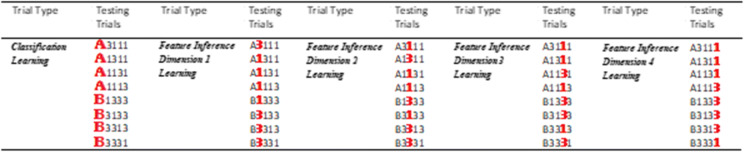
The abstract structure for all summary learning trials in Experiment 2

*Note.* Queried category labels/features for a given test case are in bold red and had a clear, unambiguous correct answer in the category summary

### Materials and methods

#### Participants

Forty-eight Cardiff University students participated for course credit or payment.

#### Materials and procedure

Before the testing trials, there were a series of training trials with the category summary present where participants received feedback on their responses. This summary learning task was based on the eight ordinary category instances in Table 1 (excluding the typical and atypical instances for each category) and included eight classification trials and 32 feature inference trials (Table 3). Each individual feature of the included instances was queried, and participants received feedback for both the classification and feature inference trials on whether their answer was correct. Participants could look at each feedback screen for as long as they wanted and clicked the mouse to continue to the next trial. The eight instances were included as all features of those instances can be unambiguously inferred (when the typical and atypical instances are excluded) and only these eight instances were present in the category summary on the screen during the feedback learning phase. After this the participants completed the same key decision-making tests as in Experiment 1 and the classic paradigm tests of standard effects including premise typicality questions at the end of the experiment. Finally, the category labels (Dreton/Rilbar) were reduced from two to one syllable (Thab/Lork) to make them easier to process. All other methodological aspects were the same as in Experiment 1.

### Results and discussion

**Fig. 7:**
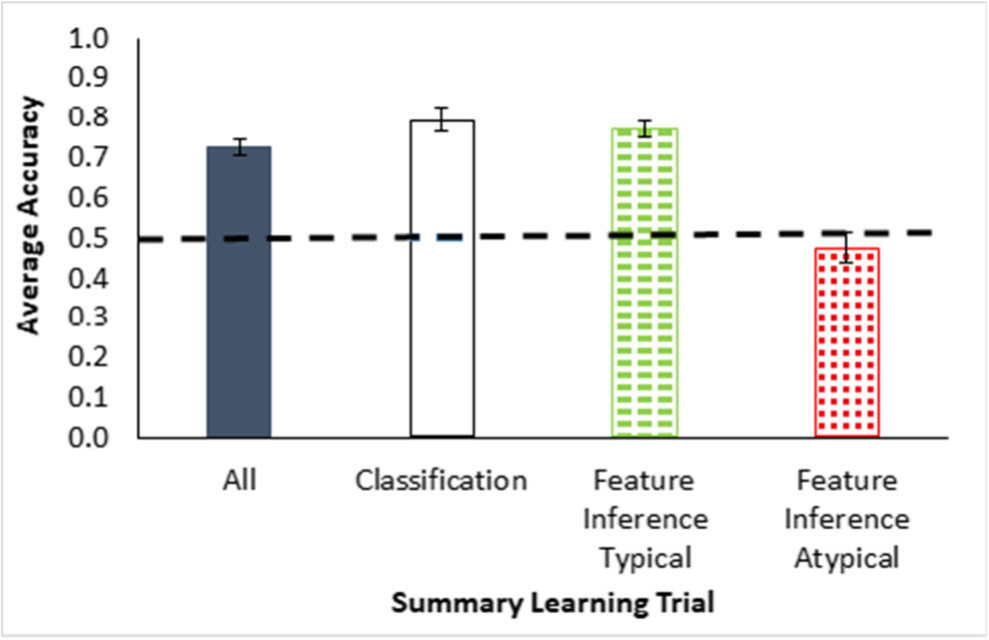
Average accuracy as proportion correct averaged across all feedback learning trials (all data = dark bar), across classification trials (classification = white bar) and averaged across all four blocks of feature inference training trials grouped by trial type (typical = green dashes, atypical = red dots) for Experiment 2. The large dashed line is a reference for two-option chance responding. Error bars show ±1 standard error. (Color figure online)

Overall accuracy on the summary learning trials (Fig. 7, first bar) was fairly high (compared with 0.5 chance responding), *t*(47) = 11.1, *p* < .001, a single-sample*t* test, *d* = 1.6, BF_10_ = 7.165e+11, suggesting that participants were attending reasonably well to all of the feature dimensions and instances. The summary learning classification trials showed good performance (Fig. 7, second bar), *t*(47) = 9.7, *p* < .001, a single-sample*t* test compared with 0.5, *d* = 1.4, BF_10_ = 9.764e+9. In addition, participants were significantly more accurate on typical feature inferences (1s for Category A and 3s for Category B in Table 3) than atypical feature inferences (the third and fourth bars in Fig. 7), *t*(47) = 6.1, *p* < .001, *d* = 0.9, BF_10_ = 81931.120, and thus showed an effect of typicality across multiple dimensions. So, in terms of necessary prerequisites for an assessment of premise typicality, participants showed good engagement with the category structure.

**Fig. 8:**
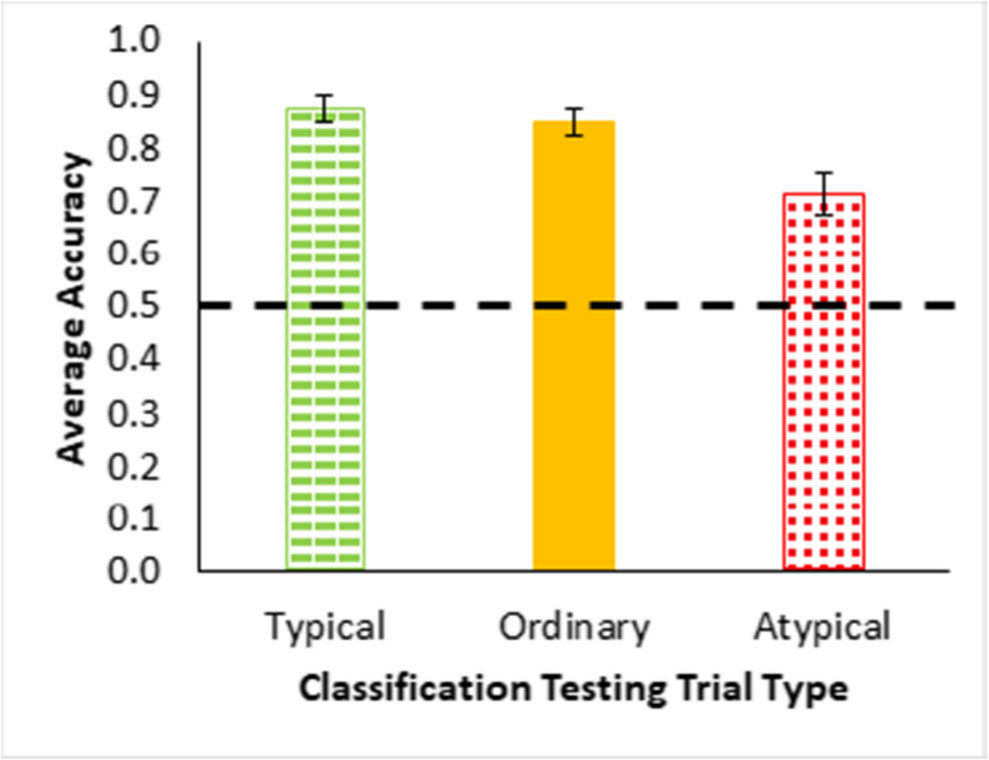
Averaged accuracy as proportion correct averaged across all blocks of classification testing trials (see Table 1) for Experiment 2, grouped by trial type—typical = green dashes, ordinary = yellow, atypical = red dots. The dashed line is a reference for two-option chance responding. Error bars show ±1 standard error. (Color figure online)

**Fig. 9:**
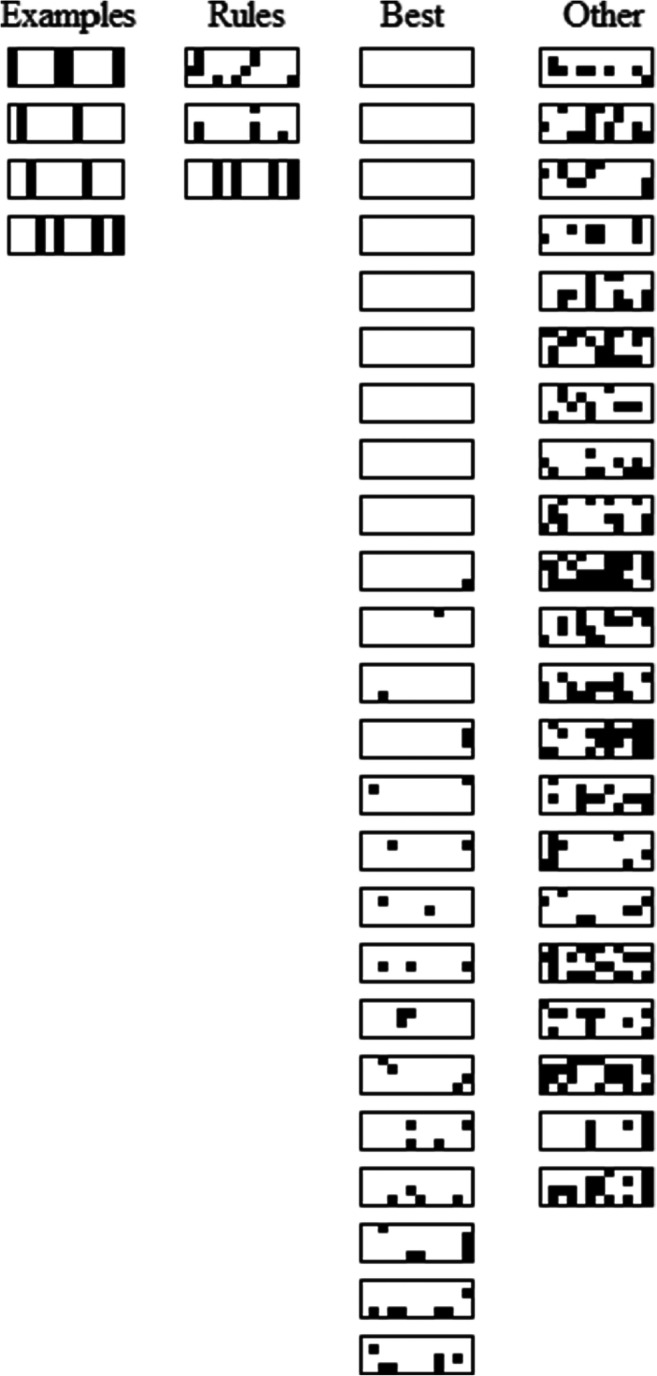
Error diagrams showing performance of each individual participant over all classification testing trials in Experiment 2. Instances are arranged in columns (ordered as in Table 1) and testing blocks are arranged in rows. See main text for details. Black dots = incorrect answers, white “dots” = correct answers. Error patterns in the “examples” grouping correspond to unidimensional rules, shown in order with a dimension one rule at the top and dimension four rule at the bottom. The “rules” grouping has apparent suboptimal dimensional rule users, the “best” group includes high accuracy performers, and the “other” group has the remaining participants that used various other strategies

The classification test results for the ordinary, typical and atypical instances were without feedback and show a typicality effect (Fig. 8) with higher accuracy for more typical than less typical instances, *F*(1.5, 68.8) = 13.874, *p* < .001, ${\eta}_{partial}^2$ = 0.228. (Note, Greenhouse–Geisser correction was applied to this single factor ANOVA, BF_10_ = 3560.330.) The feature inference feedback trials and the classification testing trials together show sensitivity to the typicality structure of the categories across dimensions. Note that participants were especially poor on responding to feature inferences for the atypical features, suggesting that they were predisposed to respond with typical features and further validating the finding of a typicality effect. In addition, this experiment fixed the potential problem in the previous experiment that some participants were seemingly attending to only one dimension (as indicated by the error diagrams for Experiment 1 in Fig. 6). Confirming this reduction in dimensional rule use, the error diagrams in the current experiment (Fig. 9) showed only 6% of participants apparently using dimensional rules. So, these results indicate that participants reasonably satisfied these additional requirements for assessing premise typicality as well as eliminating an explanation for its absence in Experiment 1.

**Fig. 10:**
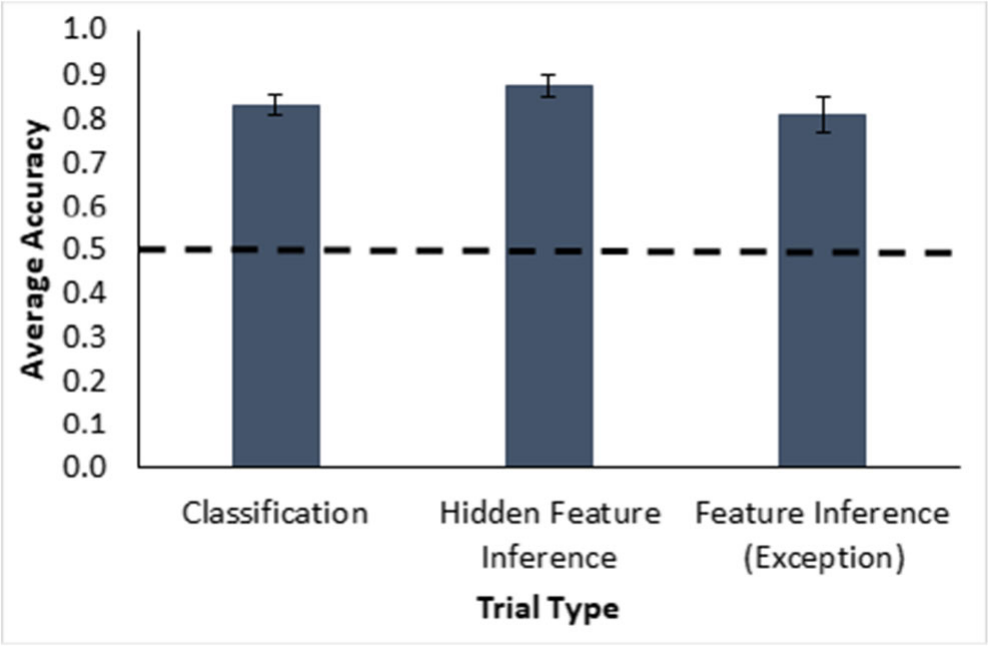
Average accuracy as proportion correct for the classification, hidden feature inference, and feature inference (exception) testing trials (see Table 2) in Experiment 2. The dashed line is a reference for two-option chance responding. Error bars show ±1 standard error

The hidden feature inference trials (see Fig. 10, middle bar) showed good attachment of the hidden features to the typical and atypical instances, *t*(47) = 14.2, *p* < .001, a single-sample*t* test, *d* = 2.0, BF_10_ = 4.092e+15. Additionally, classification performance across all testing blocks (Fig.10, left bar) was good, *t*(47) = 14.3, *p* < .001, a single-sample*t* test, *d* = 2.1, BF_10_ = 5.901e+15, as was the feature inference on exception features *t*(47) = 7.8, *p* < .001, a single-sample*t* test, *d* = 1.1, BF_10_ = 1.937e+7 (see Fig. 10, right bar). Note that the exception feature inference trials were based on the exception features of the atypical instances with their hidden features present. This is compared with the poor learning performance on feature inferences of atypical features for instances without a hidden feature, suggesting that the presence of the hidden features on a trial improves atypical feature inference. Overall, participants showed high levels of engagement with the category summary as a needed prerequisite for an assessment of premise typicality.

**Fig. 11:**
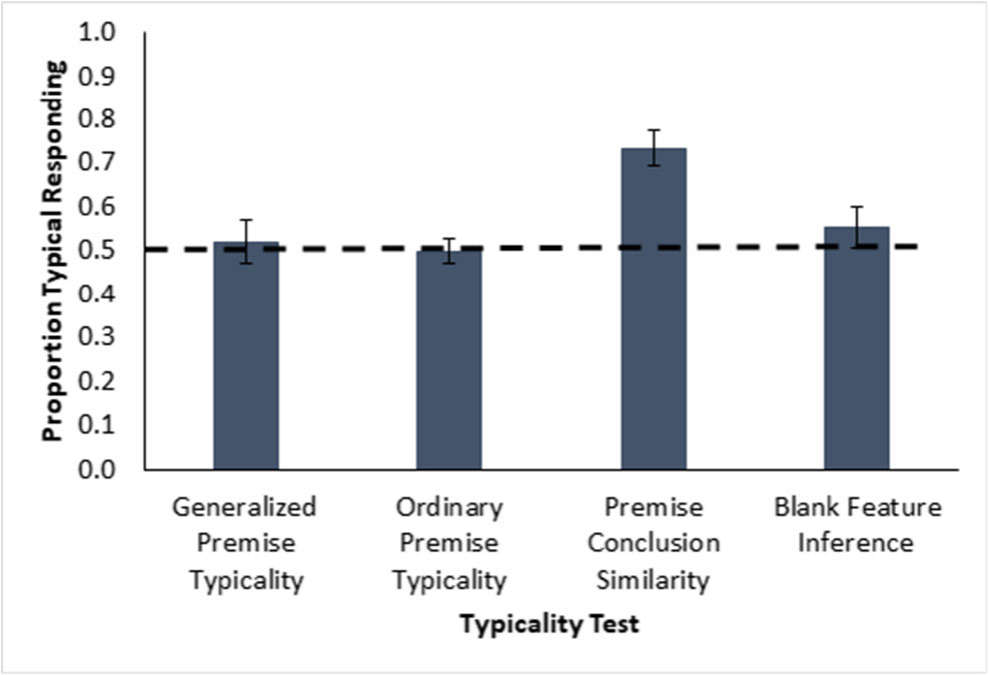
Average proportion of typical hidden feature responding for generalized and ordinary premise typicality, premise conclusion similarity, and blank feature inference in Experiment 2(Table 1). The dashed line is a reference for two-option chance responding. Error bars show ±1 standard error

Despite good attachment of the hidden features to the typical and atypical instances and sensitivity to the typicality structure of the category, no premise typicality occurred (Fig. 11) on any of the three kinds of tests where it might have plausibly occurred: not on generalized premise typicality trials, *t*(47) = 0.4, *p* = .681, single-sample*t* test, *d* = 0.1, BF_10_ = 0.170, or ordinary premise typicality trials, *t*(47) = 0, *p* = 1, single-sample*t* test, *d* = 0, BF_10_ = 0.157 (note that the average proportion was exactly 0.50). And the blank feature inference trials where only the label was present also did not show the effect, *t*(47) = 1.1, *p* = .280, single-sample*t* test, *d* = 0.2, BF_10_ = 0.275. The best participants in Fig. 9 showed neither generalized premise typicality, *t*(23) = 0.4, *p* = .689, single-sample*t* test, *d* = 0.1, BF_10_ = 0.231, nor ordinary premise typicality, *t*(23) = −1.6, *p* = .135, single-sample*t* test, *d* = −0.3, BF_10_ = 0.613. Participants showed no preference for generalizing the hidden feature from the typical instance compared with the atypical instance when similarity of the test instance to the typical and atypical instances was the same, and the Bayesian statistics show significant support for this lack of a difference. So, as in Experiment 1, no premise typicality effects occurred. However, a significant premise conclusion similarity effect occurred (Fig. 11, third bar), *t*(47) = 5.6, *p* < .001, single-sample*t* test, *d* = 0.8, BF_10_ = 16313.461. And as in the previous experiment, the proportion of typical responding was significantly higher for premise conclusion similarity than for generalized premise typicality, *t*(47) = 3.3, *p* = .002, within-participants*t* test, *d* = 0.5, BF_10_ = 15.43. So, a premise typicality *like* effect occurred here, too, but plausibly due only to similarity rather than typicality.

The classic paradigm tests produced a significant effect of premise typicality based on a difference in rated argument strength for the typical premise (mean = 4.73) greater than the atypical premise (mean = 4.06), *t*(47) = 2.5, *p* = .014, *d* = 0.4, BF_10_ = 2.822. This again indicates that there is no defect in the participant population to attribute the lack of premise typicality to.

## General discussion

In these two experiments we evaluated the premise typicality effect from the categorical induction paradigm (Carey, 1985; Hayes et al., 2010; Osherson et al., 1990; Rhodes et al., 2008; Rips, 1975) using the perceptual categorization paradigm in order to better control for the similarity of test cases to typical and atypical category instances. To do this, we used a category structure that had a typicality gradient with “hidden” features (shown by a cutout view of the interior of some stimuli) attached to typical and atypical instances. Testing instances that were equally similar to the typical and atypical instances assessed premise typicality as a preference for the feature attached to a typical category instance over the feature attached to an atypical instance. Both experiments presented a visual summary of category instances on every testing trial (Fig. 1). Experiment 1 was a pure decision-making task with no feedback, but Experiment 2 started with a training phase during which participants were given corrective feedback over a series of classification and feature inference trials for instances in the summary. Neither experiment found premise typicality effects, with significant Bayesian support for its absence, but both found premise conclusion similarity effects, a preference for the hidden feature attached to the more similar instance.

The original intent of this research was to establish analogues of key categorical induction effects, especially premise typicality, to be able to assess the representational basis of these effects using exemplar and prototype models (Homa et al., 1981; Kruschke, 1992; Medin & Schaffer, 1978; Nosofsky, 1986; J. D. Smith, 2002). However, the present experiments have not found premise typicality effects. So, having failed in its initial intent, what are the conceptual implications of this research? We do not question the existence of this widely replicated effect in the categorical induction paradigm per se (Carey, 1985; Hayes et al., 2010; Osherson et al., 1990; Rhodes et al., 2008; Rips, 1975) but rather its basis in category typicality as distinct from instance similarity, when these are carefully specified in the perceptual categorization paradigm and are distinct from influences of specific semantic/causal knowledge for real world categories.

Given that typicality effects have been widely demonstrated using perceptual categories, it seems reasonable to expect premise typicality effects if these are fundamentally based on typicality. Below we consider possible conceptual and methodological reasons for the observed absence of premise typicality effects in our results using perceptual categories and discuss their implications. Most of these potential explanations are based on the differences between the perceptual categorization and categorical induction paradigms.

Perhaps the most notable difference between the paradigms is in the intended role of prior category knowledge. In contrast to both paradigms, some research (e.g., McRae et al., 1997) has assessed prior knowledge of both real-world categories and their attributes. But a lot of research on the mechanisms of learning and reasoning attempts to distinguish the influences of these mechanisms from prior knowledge by restricting the applicability of that knowledge: For example, assessments of causal reasoning commonly elicit judgements about the strength of relationship between a light and a button (e.g., Greville et al., 2013) where prior knowledge of lights and buttons allows the affordance of a possible relationship without requiring it or placing much constraint on its strength. Similarly, assessments of human analogues of animal associative learning phenomena like blocking use tasks such as disease diagnosis with real symptoms (e.g., stuffy nose), but blank outcomes like disease A (e.g., Kruschke & Blair, 2000), so prior knowledge implies learnability without determining its form. The categorical induction paradigm has used real categories which intentionally allow a great deal of prior knowledge to potentially bear on the task, e.g., robins as a subcategory of birds and the many associated attributes these have. However, prior knowledge of the to-be-inferred attributes (e.g., property X) is usually chosen to minimize prior specific knowledge of the attributes themselves to facilitate the assessment of reasoning processes rather than factual knowledge per se. Nonetheless responding in this paradigm is in the context of prior knowledge of the categories. But characterizing the influence of this knowledge is complex making experimental control at best challenging. In contrast the perceptual categorization paradigm has intentionally tried to minimize the influence of prior knowledge even more by specifying both novel categories and relations among feature attributes, and so allowing somewhat greater experimental control. As a result, there are two contrasting possible reasons why the present experiments failed to produce premise typicality effects: One is that the perceptual categories used may not have done a good enough job in terms of eliminating influences of prior knowledge, and this residual knowledge is in some way responsible for the absence of the effect. The second is that the perceptual categories have done too good a job in terms of eliminating influences of prior knowledge, and that this elimination of context is responsible for the absence of the effect.

While participants in the present experiments very likely had prior knowledge of rocket ships and their common attributes (e.g., long and thin, pointy at one end in the direction of travel, flatter at the other end that generates thrust), there is no reason to think they had any knowledge of the rocket categories used here (e.g., Dreton and Rilbar; Fig. 1). While prior familiarity with rocket ships and how their attributes vary likely helped participants process the rockets in the two categories, lack of prior knowledge of the categories themselves gives little basis for expecting which features should go together. And while prior knowledge likely includes that rockets have internal features, this limited knowledge does not seem to substantially constrain the plausible forms of those features (e.g., as in Fig. 1). As such, the limited prior knowledge that participants brought to bear on the task does not seem to plausibly explain the *lack* of a preference for one hidden feature over another. If anything, the opposite seems more plausible: These tasks may have been a somewhat unfair test of premise typicality in that premise typicality potentially arises out of prior knowledge.

The contrasting explanation for the lack of premise typicality in the present experiments then might be the lack of relevant semantic knowledge in memory (see Kumar, 2021, for a current review of theories of semantic representation). As such, the limited prior knowledge that participants had about the internal attributes of rockets provided no basis for preferring one hidden feature over the other, so participants may just have guessed on the key tests of premise typicality. Nevertheless, the conceptual specification of premise typicality is not in terms of complex semantic knowledge per se but specifically in terms of typicality: attributes of more typical instances should generalize better all else being equal.

After controlling similarity in perceptual categories, premise typicality effects either exist, suggesting they are based on typicality, or they do not, suggesting they are not fundamentally based on typicality. More specifically, either the present experiments have not set up the appropriate conditions, in which case the key question is what are those conditions, or premise typicality as distinct from similarity does not exist in this paradigm, in which case the key question is why not?

The present experiments have eliminated some but by no means all possible methodological reasons for the observed absence of premise typicality effects in these perceptual categorization experiments. The design and results of Experiments 1 and 2 plausibly eliminate mundane explanations in terms of lack of participant engagement or attention in that accuracy on classification and hidden feature inference was reasonably good. And both experiments produced apparent typicality effects, with Experiment 2 convincingly eliminating the rule use explanation for the typicality effect in Experiment 1. It seems reasonable to have looked for premise typicality using a summary presentation of categories given the prevalence of this methodology in perceptual categorization research (Griffiths et al., 2012; Johansen et al., 2015; Murphy & Ross, 1994, 2010; Yamauchi & Markman, 2000; Yamauchi & Yu, 2008), and most categorical induction research uses summarized information. Further, the summary presentation seems highly conducive to participants noticing which features are typical and which are atypical, as supported by typicality effects in both of the current experiments, even though the numbers of category instances and features within those instances are reasonably small. Nevertheless, it is possible that premise typicality requires fully internalized category representations to produce “real” rather than apparent typicality effects—for example, as a result of better memory for typical features than atypical. Another possibility is that premise typicality is an emergent property of an entire knowledge hierarchy of categories within categories in a way that using typical and atypical instances of simple perceptual categories does not capture. Nevertheless, attempts to disambiguate the influences of similarity from those of typicality on feature inference in perceptual categories are warranted, in particular in the context of key theoretical differences between the prototype and exemplar theories of category representation. Specifically, prototype theory seems to intrinsically distinguish typicality from instance similarity while exemplar theory does not.

The alternative possibility is that premise typicality effects really do not exist for perceptual categories as distinct from instance similarity effects. The present results are consistent with this possibility but do not definitively establish it, as discussed in the previous paragraph. More controversially, it is possible that premise typicality effects in the classic categorical induction paradigm (Carey, 1985; Hayes et al., 2010; Osherson et al., 1990; Rhodes et al., 2008; Rips, 1975), are based on (possibly subtle) differences in similarity rather than typicality per se. But regardless, attempts to map effects between these two paradigms seem likely to be fruitful because of the common questions about their underlying representational basis. In conclusion, facilitating attribute inference is central to the functionality of categories, which emphasizes key questions: what are the category representations underlying attribute inference for category instances? More specifically, are influences of instance typicality and similarity on attribute influence meaningfully distinct? The present results suggest they are not, but more research is needed.

## Figures and Tables

**Table 4:**
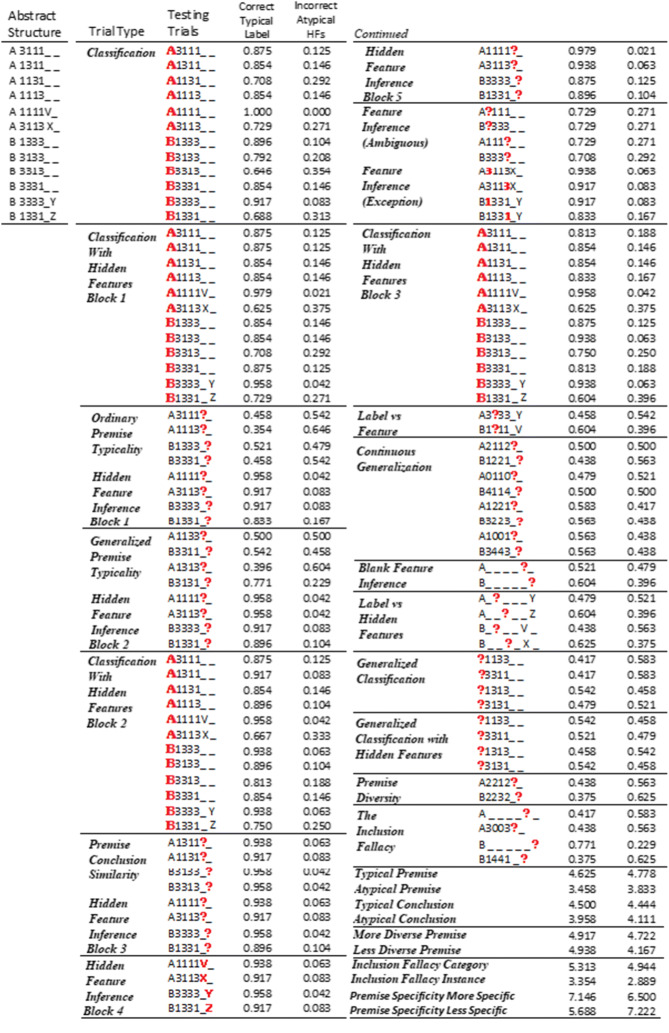
Experiment 1’s abstract category structure and full list of testing blocks and trials with average response proportions

*Note.* Category labels/features in bold red for a given test case were queried and had an unambiguous correct answer in the category summary. Question marks in test cases indicate a queried feature that did not have a clear correct answer, i.e., with no single best match in the category structure

**Table 5:**
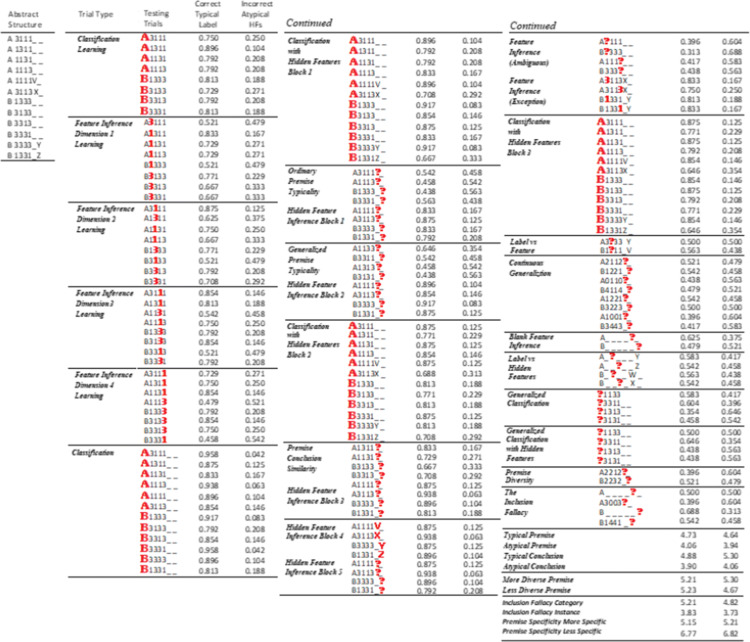
Experiment 2’s abstract category structure and full list of learning and testing blocks and trials with average response proportions

*Note.* Category labels/features in bold red for a given test case were queried and had an unambiguous correct answer in the category summary. Question marks in test cases indicate a queried feature that did not have a clear correct answer (i.e., with no single best match in the category structure)

## Appendix A: Full specification of all trials in Experiments 1 and 2 with average response proportions for each trial.

For each experiment summary, the abstract category structure is in the left, top corner of the tables (Tables 4 and 5). The next column is the descriptor for the construct that each block was training/testing followed in the next column by the abstract structure of the trials. The next two columns contain the average response proportions over all participants with the first column showing averaged abstract correct/typical/label-based responding depending on the trial. If the trial had a unique correct answer in the category summary or given in the learning task, then the first column was a measure of responding with that correct answer. If the trial was querying either a hidden feature for an instance other than the typical and atypical of each category or a non-hidden feature and there was no correct answer (as in there was no exact match or multiple matches in the category summary or learning task) column one was a measure of responding with the typical feature. If the trial compared the effects of the label against another stimulus component, the feature typical of the category the label was denoting is considered to be label-based responding therefore, for these trials, column one represents the proportion of participants responding with the label consistent option. The second column shows the averaged abstract incorrect/atypical/hidden feature-based responding, contrary to the responding displayed in the first column. It shows responding with the incorrect answer, the atypical feature or the feature typical of the category denoted by the hidden/nonhidden feature/s, respectively. For each trial in the table, the letter/number/symbol in bold red was queried. Bold red letters/numbers indicated that for a queried label/feature there was a correct answer in the category summary or learning task. A red question mark indicated the label/feature that was queried had no basis in the category summary or learning task for responding with any one answer over the other or there were two answers that were consistent with the information provided on that trial.

## Appendix B: Additional testing trials included in Experiments 1 and 2

Experiments 1 and 2 included additional testing trials that were not central to the key assessments of premise typicality. These are described in detail below and were intended to provide additional clarification and constraints for models. The average response proportions across participants are in Appendix A.

The current experiments included feature inference (ambiguous) testing trials that matched two instances in the category summary, the typical instance and an ordinary instance and these predicted different features as a response. For example, the instance A?111 has the same last three features as both the typical instance A1111 and the ordinary instance A3111. So, based on a match to a single category instance, both 1 and 3 are possible responses; however, a 1 feature is the more typical feature for Category A, so a 1 feature response potentially corresponds to a typicality effect.

Exploratory trials in these experiments evaluated the relative influence that each part of the stimulus had on responding—category labels, nonhidden features and hidden features—by pitting these against each other. In the “label versus feature” trials the category label from one category was combined with the typical features of the other category and participants were queried on a missing nonhidden feature. Similarly, the “label versus hidden feature” trials contrasted a feature inference response consistent with the category label (the typical nonhidden feature for that category) to the response consistent with the hidden feature (the atypical nonhidden feature for the category denoted by the category label).

Another common effect in categorical induction is premise diversity in which the conclusion is judged as stronger when the premises of an argument are diverse in their coverage of a category. Experiments 1 and 2 tested premise diversity by adding a hidden feature to one additional instance in each category in the category summary (specifically the A1311 and B3133 instances) that was typical and atypical, respectively. Therefore, Category A had a less diverse set of instances with the typical hidden feature (A1111 and A1311) whereas Category B had a more diverse set of instances with the atypical hidden feature (B1331 and B3133). The test instances were A2212 and B2232 which were equally similar to the typical and atypical instances for each category. These are continuous instances, as the 2 value relates to the feature dimensions on a continuum from 0 to 4 (e.g., rocket ship wing width).

The inclusion fallacy is a further categorical induction effect which occurs when a conclusion that covers a category is judged stronger than a conclusion that is a member of that category. Experiments 1 and 2 attempted to test the inclusion fallacy via a blank feature inference trial (a trial with no feature information present on the screen, only a category label is presented) and a specific category instance for each category. The specific category instances were A3003 and B1441. Continuous instances were used as all noncontinuous instances had been used in other testing trials and may have had associations separate from the inclusion fallacy. In addition, Experiments 1 and 2 also included generalization classification trials which queried the category label for the four instances that were not present in the category summary/not trained during the training phase (A1133, B3311, A1313, B3131). These trials potentially tested for dimensional rule use.

## Appendix C: All classic paradigm categorical induction questions used in Experiments 1 and 2(from Hayes et al., 2010)

Premise Typicality Question 1:

Sparrows have property X / Therefore / Geese have property X

Premise Typicality Question 2:

Penguins have property X / Therefore / Geese have property X

Conclusion Typicality Question 1:

Vultures have property Y / Therefore / Sparrows have property Y

Conclusion Typicality Question 2:

Vultures have property Y / Therefore / Quail have property Y

Premise Diversity Question 1:

Lions have property Z / Mice have property Z / Therefore / Mammals have property Z

Premise Diversity Question 2:

Lions have property Z / Tigers have property Z / Therefore / Mammals have property Z

Inclusion Fallacy Question 1:

Crows have property A / Therefore / Birds have property A

Inclusion Fallacy Question 2:

Crows have property A / Therefore / Ostriches have property A

Premise Specificity Question 1:

Birds have property B / Therefore / Sparrows have property B

Premise Specificity Question 2:

Animals have property B / Therefore / Sparrows have property B
